# Editorial: Environmental Enrichment: Enhancing Neural Plasticity, Resilience, and Repair

**DOI:** 10.3389/fnbeh.2019.00075

**Published:** 2019-04-16

**Authors:** Amanda C. Kentner, Kelly G. Lambert, Anthony J. Hannan, S. Tifffany Donaldson

**Affiliations:** ^1^School of Arts & Sciences, Health Psychology Program, Massachusetts College of Pharmacy and Health Sciences, Boston, MA, United States; ^2^Department of Psychology, University of Richmond, Richmond, VA, United States; ^3^Melbourne Brain Centre, Florey Institute of Neuroscience and Mental Health, University of Melbourne, Parkville, VIC, Australia; ^4^Department of Anatomy and Neuroscience, University of Melbourne, Parkville, VIC, Australia; ^5^Developmental and Brain Sciences, Department of Psychology, University of Massachusetts Boston, Boston, MA, United States

**Keywords:** environmental enrichment (EE), neuroprotection, rehabilitation, standardization, reproducibility, animal welfare, translational

## Introduction

Up until the middle of the twentieth century, neuroscience dogma emphasized the fixed, immutable nature of the brain. In the 1960s, however, in what was arguably one of the most revolutionary research programs in neuroscience, a team of researchers at UC Berkeley generated data revealing the dynamic characteristics of mature neural systems. Working with Edward Bennett, David Krech, and Mark Rosenzweig, Marian Diamond (1926-2017) documented the neuroanatomical changes in rats exposed to enriched environments—findings that threatened prior notions of brains being “fixed in stone” (Bennett et al., 1964). In an interview (with KL) several years ago, Diamond described her experience presenting these unexpected results for the first time at a professional conference. After delivering her talk, she heard an audience member shout, “young lady, that brain cannot change!”

Although Diamond confirmed that she had replicated the findings, perceptions about the changing nature of the brain were initially met with resistance and skepticism (personal communication, October, 2009). Not to be dissuaded, Diamond continued her neuroanatomical explorations, verifying that rats exposed to novel and complex environments experienced equally complex neural changes, especially in the cerebral cortex. Although hints of neural plasticity had been introduced by individuals such as Charles Darwin and Donald Hebb prior to Diamond's research, she has been credited for providing tangible evidence of neuroplasticity. One of her colleagues at the University of California at Berkeley, George Brooks, wrote in her obituary, “Dr. Diamond showed anatomically, for the first time, what we now call plasticity of the brain” (Sanders, 2017). She was also remembered as the neuroscientist who gave a new meaning to the idea of “use it or lose it,” as she shattered previous notions of a static unchangeable brain that simply degenerated throughout life (Smith, 2017). Through her pioneering work with enriched environments, Marian Diamond most certainly enriched the field of neuroscience, as the notion of neuroplasticity has become a corner stone of contemporary neuroscience. Moreover, her research has inspired many other important investigations into the translational nature of environmental enrichment.

Dedicated to Dr. Diamond, the current Research Topic focuses on environmental enrichment in terms of neuroprotection/rehabilitation and the use of this system in the laboratory to promote species-typical behavior and generalizable, rigorous investigation into the effects of experience on behavior and physiology.

## Standardization and Environmental Enrichment

Concerns that environmental enrichment may increase variability in experimental endpoints, affecting reproducibility, have impeded the expansion of its use in research. Data described by Bailoo et al. demonstrate that this may be an unfounded assumption. The coefficient of variation across housing conditions did not differ on measures of behavior, growth, or stress physiology. Additionally, the more enrichment provided, the more engagement mice had with their environment. Similarly, Neal et al. report that enhanced social environments facilitate affiliative behaviors and appear to promote problem solving behaviors. Together, this underscores the importance of enrichment for healthy species-typical behaviors.

## Physiological and Neurobehavioral Effects of Environmental Enrichment

Morano et al. demonstrated that removing female rats from environmental enrichment mimics an experience of “loss,” leading to increased passive coping behavior, hyperphagia, and evidence of HPA axis dysregulation. These behavioral and physiological changes are associated with an array of psychiatric disease states, suggestive of the utility of this model for understanding loss-related dysfunctions. In contrast, Cutuli et al. review the effects of enrichment exposure across critical periods of development, particularly on parental care and offspring outcomes. Moreover, they provide a specific emphasis on the importance of pre-reproductive vs. post-reproductive experiences and transgenerational effects, highlighting some of their own research findings.

While environmental enrichment can be stressful to some male animals, McQuaid et al. show that post-weaning enrichment increases demonstrations of social behavior, buffered corticosterone, and central cytokine levels, and elevated brain-derived neurotrophic factor in the prefrontal cortex of male mice, following stressor exposure in adulthood.

## Environmental Enrichment and Clinical/Neuroprotection

Environmental enrichment has been demonstrated to induce therapeutic effects in a wide range of preclinical models of neurological and psychiatric disorders. Various articles in this Research Topic have addressed this important issue, providing new insights into molecular and cellular mechanisms of experience-dependent plasticity, and identifying novel candidate targets for enviromimetics (Nithianantharajah and Hannan, 2006).

The first demonstration that EE can be beneficial in a genetic model of a human disorder utilized a transgenic mouse model of Huntington's disease (Van Dellen et al., 2000). Zajac et al. have now demonstrated that one of the molecular effects of EE, even when delivered for a relatively short period (2 weeks), is transcriptional modulation of components of the serotonergic system. Furthermore, these investigators compared EE with an exercise (voluntary wheel running) intervention and found key differences between these two environmental interventions, as well as interesting region-specific and sexually dimorphic effects on gene expression. Finally, they were able to demonstrate functional consequences of the EE effect, via behavioral pharmacology.

The effects of EE on a different mouse model, with haploinsufficiency in the brain-derived neurotrophic factor (BDNF) gene, were described by Grech et al. The authors use a model in which they combine the two negative factors (heterozygosity for the BDNF null allele and chronic administration of the stress hormone corticosterone) to create a “two hit model” of relevance to specific neurodevelopmental and psychiatric disorders. Interestingly, these authors also found sexually dimorphic effects of EE, and were able to correlate their behavioral data with expression levels of the neurotrophin receptor TrkB (and its signaling, via quantification of phospho-TrkB) and specific NMDA receptor subunits, reinforcing the evidence for a role of neurotrophic and glutamatergic signaling in mediating the therapeutic effects of EE.

Another key aspect of EE, which has been often overlooked, is enrichment of the nest conditions in rodent models. Mason et al. have addressed this via a form of nesting enrichment called “closed nest boxes.” This form of nesting enrichment was shown to induce beneficial effects in a model of neonatal hypoxia-ischemia. Interestingly, molecular correlates included BDNF and glial-derived neurotrophic factor (GDNF), and sexually dimorphic effects were observed, which has become a recurring theme in EE studies.

Finally, the combination of EE with other therapeutic approaches is an area that has been under-explored and has substantial translational potential. Bhaskar et al. have combined EE with deep-brain stimulation (DBS) and found that the combination increased anxiolytic effects relative to the same DBS intervention performed in standard-housed animals. Combining EE with other interventions, both pharmacological (e.g., enviromimetics; Nithianantharajah and Hannan, 2006) and non-pharmacological (in this case a model of medical device), could reveal additive and synergistic effects that greatly enhance therapeutic efficacy in a wide range of neurological and psychiatric disorders.

## Environmental Enrichment and Aging

Environmental enrichment may help protect against the effects of cognitive decline. As discussed in Leon and Woo, stimulation offered through sensory enhancement may also offset the effects of aging on not only the brain, but on multiple body systems significantly impacted by time.

Rapley et al. measured C-type Natriuretic Peptide (CNP) to determine its expression across normal aging and its interaction with the environment. Enrichment housing transiently increased CNP availability in young, but not older rats. This may be suggestive of age-related losses in sensitivity to environmental stimulation.

## Translation

The benefits of environmental enrichment are reported in preclinical settings with little focus on translation. McDonald et al. describe many obstacles to application of environmental enrichment paradigms to patients experiencing stroke. The work highlights the variation between animal and clinical models of enrichment, suggesting that greater alignment is required to improve translation.

## Future Directions

The potential for the environment to influence behavior has a long history, and subsequent work implicating correlative brain changes were pioneered by Dr. Marian Diamond. The contributions to this Research Topic offer findings regarding enrichment promoting healthy species-typical behavior in the lab and reversing insult. It is the hope of the editors that this evidence will support wider adoption of enrichment in normal housing protocols.

Moreover, it is important to devote attention to the aspects of enrichment in preclinical settings that promote plasticity, to enable the recreation of these components in clinical settings. As McDonald et al. intimate, this may very well present an opportunity to translate successful interventions to clinical populations. Additional work that advances understanding of the mechanisms underlying the benefits of enrichment in healthy and injured/adverse populations is also warranted (Kentner et al., 2019).

We also hope that further work is accomplished that delineates *when* and *how long* enrichment is needed for activating benefits and promoting plasticity across the lifespan. Since many disease states show gender disparities, another line of inquiry should include a focus on biological sex differences in enrichment paradigms. Enrichment shows distinct sex-specific benefits following early insult (Grech et al.; Mason et al). Few researchers are attending to the intergenerational benefits of enrichment (Cutuli et al.) and variation across the age axis, so continued efforts in these areas are also necessary.

After more than six decades of work on environmental enrichment, we applaud the advances in understanding relevant biological parameters and key mechanisms, and emphasize its potential for adaptation to clinical populations, across a broad spectrum of neurological and psychiatric disorders.

## Author Contributions

All authors contributed to the writing of the editorial. AK and SD facilitated the concept, design, and overall development of the manuscript.

### Conflict of Interest Statement

The authors declare that the research was conducted in the absence of any commercial or financial relationships that could be construed as a potential conflict of interest.
